# Prolonged hemophagocytic lymphohistiocytosis syndrome as an initial presentation of Hodgkin lymphoma: a case report

**DOI:** 10.1186/1752-1947-2-367

**Published:** 2008-12-04

**Authors:** Kathryn Chan, Eric Behling, David S Strayer, William S Kocher, Scott K Dessain

**Affiliations:** 1 Cardeza Foundation for Hematologic Research and Kimmel Cancer Center, 1015 Walnut St., Philadelphia, PA, USA; 2 Department of Medical Oncology, Benjamin Franklin House, Suite 314, 834 Chestnut St., Philadelphia, PA, USA; 3 Department of Pathology, Anatomy and Cell Biology, Pavilion Building, Suite 301, 125 S. 11th St., Thomas Jefferson University, Philadelphia, PA 19107, USA; 4 Lankenau Institute for Medical Research, Room 227, 100 Lancaster Avenue, Wynnewood, PA 19096, USA

## Abstract

**Introduction:**

Hemophagocytic lymphohistiocytosis is an immune-mediated syndrome that typically has a rapidly progressive course that can result in pancytopenia, coagulopathy, multi-system organ failure and death.

**Case presentation:**

A 57-year-old Caucasian woman was referred in fulminant hemophagocytic lymphohistiocytosis, with fever, pancytopenia, splenomegaly, mental status changes and respiratory failure. She was found to have stage IV classical Hodgkin lymphoma, in addition to Epstein-Barr virus and cytomegalovirus viremia. Her presentation was preceded by a 3-year prodrome consisting of cytopenia and fever that were partially controlled by steroids and azathioprine.

**Conclusion:**

Fulminant hemophagocytic lymphohistiocytosis may follow a prodromal phase that possesses features suggestive of a chronic form of hemophagocytic lymphohistiocytosis, but which may also resemble immune cytopenias of other causes. A diagnosis of hemophagocytic lymphohistiocytosis should be considered in the setting of chronic pancytopenia.

## Introduction

Hemophagocytic lymphohistiocytosis (HLH) is a syndrome characterized by fever, hepato-splenomegaly, lymphadenopathy, pancytopenia, rash, and hemophagocytosis by non-malignant macrophages [1,2]. Laboratory findings characteristic of this disease include hypertriglyceridemia, hyperferritinemia, hypofibrinogenemia and liver function test abnormalities. The symptoms of HLH are typically rapidly progressive, often resulting in death from hemorrhage, multi-system organ failure, or infection. Survival from HLH requires prompt recognition of the syndrome, correction of its underlying cause, and HLH-specific therapies such as etoposide [3].

HLH occurs in both inherited and acquired forms. Inherited forms have been attributed to defects in perforin function and other intracellular pathways required for the release of cytolytic granules by NK cells and cytotoxic T-lymphocytes [2]. In its acquired forms, HLH has been associated with infections, such as Epstein-Barr virus (EBV) and cytomegalovirus (CMV), inflammatory diseases, such as juvenile rheumatoid arthritis, and malignancies, such as T-cell non-Hodgkin lymphoma and Hodgkin lymphoma (HL) [2,4]. In HLH, an apparent loss of restraint of the function of normal histiocytic cells is correlated with the elaboration of high levels of interferon-γ by activated CD8+ T-cells and TNF-α and IL-6 by activated macrophages [5].

Acquired forms of the disease typically follow a rapid course. In a series of six cases associated with Epstein-Barr infection, all of the patients died within 3 months of the initial onset of symptoms [6]. In most cases associated with HL, the first symptoms suggestive of HLH preceded death or definitive therapy by only 1 to 2 months [7-11].

## Case presentation

A 57-year-old Caucasian woman was admitted to a hospital in Philadelphia, PA, USA in October, 2006 for persistent fever and pancytopenia following debridement of a buttock abscess.

Three years before her admission, she had rectal bleeding and was found to have a platelet count of 65 × 10^9^/liter (laboratory reference values are given in Table 1). Upper gastrointestinal (GI) workup revealed gastric ulcers with an associated *Helicobacter pylori *infection. She received appropriate therapy, but developed a rash and a decline in her platelet count to 30 × 10^9^/liter and her hemoglobin (Hb) concentration to 80 g/liter. Bone marrow examination showed a hypercellular marrow with a myeloid left shift. Computed tomography (CT) scan revealed splenomegaly without focal lesions but no lymphadenopathy. She was treated with prednisone (1 mg/kg) for a presumed autoimmune anemia with thrombocytopenia (Evans syndrome). Her anemia corrected, but her platelet count rose to only 59 × 10^9^/liter. She continued to have cytopenias with febrile episodes. She received additional courses of steroids and was maintained on azathioprine (3.5 mg/kg/day). At one point, she had a white blood cell count (WBC) nadir < 0.5 × 10^9^/liter and was given filgrastim. Three months before her admission, she felt well and was employed full-time. Her blood counts were: WBC 1.8 × 10^9^/liter with an absolute neutrophil count of 1.386 × 10^9^cells/liter, Hb 108 g/liter, mean cell volume (MCV) 85.8 fL, platelet count 112 × 10^9^/liter.

**Table 1:** Admission laboratory studies

Test	Change	Value	Reference values
Sodium	H	126 mmol/liter	135–146 mmol/liter
Potassium		4.3 mmol/liter	3.5–5.0 mmol/liter
Chloride	L	94 mmol/liter	98–109 mmol/liter
Bicarbonate	L	21 mmol/liter	24–32 mmol/liter
BUN		7.5 mmol/liter	2.5–7.5 mmol/liter
Creatinine		62 μmol/liter	62–124 μmol/liter
Bilirubin, total	H	46 μmol/liter	3.4–21 μmol/liter
Bilirubin, direct	H	17 μmol/liter	0.0–7 μmol/liter
AST	H	78 U/liter	7–35 U/liter
ALT	H	53 U/liter	1–30 U/liter
Protein, total	L	39 g/liter	60–85 g/liter
Albumin	L	29 g/liter	32–49 g/liter
Triglycerides	H	4.2 mmol/liter	<2.3 mmol/liter
LDH	H	1068 U/liter	100–200 U/liter
Troponin		0.05 μg/liter	0.05–0.50 μg/liter
Iron		8.2 μg/dl	7.2–27.8 μg/dl
Iron binding capacity	L	32.8 μg/dl	44.8–71.6 μg/dl
Iron saturation		25%	20–55%
Ferritin	H	20,392 μg/liter	20–150 μg/liter
Haptoglobin	L	<0.6 μmol/liter	2–16 μmol/liter
White blood cell count	L	2.7 × 10^9^/liter	4–11 × 10^9^/liter
Hemoglobin	L	73 g/liter	125–150 g/liter
Hematocrit	L	0.21%	0.36–0.46
MCV		86 fl	80–99 fl
Platelets	L	15 × 10^9^/liter	140–400 × 10^9^/liter
PT	H	22.5 s	11.6–14.8 s
INR	H	1.93	0.85–1.15
PTT	H	51 s	225–33 s
Fibrinogen	L	<0.6 g/liter	2.0–4.4 g/liter
D-dimer	H	3.43 mg/liter	<0.53 mg/liter
Direct antiglobulin test		negative	

H, high value; L, low value; BUN, blood urea nitrogen; INR, international normalized ratio; PT, prothrombin time; PTT partial thromboplastin time; Reference values are institutional standards converted to SI units.

Eleven days before her referral, she was admitted to an outside hospital with temperature of 38.6°C (101.5°F) and a right buttock abscess. Her WBC was 0.5 × 10^9^/liter, Hb 75 g/liter, platelets 47 × 10^9^/liter. The buttock lesion was debrided. Bone marrow biopsy showed a hypercellular marrow with erythroid and megakaryocytic hyperplasia and clusters of atypical megakaryocytes. Her medications included decadron 20 mg q 12 hours, filgrastim, darbepoietin, albumin, imipenem, diflucan, acyclovir, protonix, folate, and vitamin B12.

### Admission studies

Examination was notable for temperature 37.7°C (100.0°F), blood pressure 126/68, respiratory rate 28, pulse 122, oxygen saturation 92% on room air. She had pallor, mild jaundice, a 2/6 systolic flow murmur, splenomegaly, a 3 × 3 cm right buttock eschar, ecchymoses on her arms and left flank, and 3+ bilateral lower extremity edema. She had no lymphadenopathy, hepatomegaly, or musculoskeletal findings. Her mental status exam was significant for a flat affect and orientation to self, but not to the current year, the hospital name, or her date of birth.

Laboratory studies (Table 1) were remarkable for pancytopenia and a coagulopathy. She had elevations in her ferritin, triglycerides, bilirubin, and liver function tests. Her haptoglobin was low, but her direct anti-globulin test was negative. Her peripheral blood smear demonstrated no spherocytic or microangiopathic changes. An antinuclear antibody (ANA) screen was also negative.

Chest CT demonstrated bibasilar patchy pulmonary consolidation, small pleural effusions, and small calcified mediastinal lymph nodes. Abdominal CT scan showed an enlarged spleen with 10 low-density, indeterminate lesions, similar lesions in the liver, bilateral renal infarcts, and a left psoas hemorrhage. Head CT and echocardiography were unremarkable.

### Hospital course

Decadron, antibiotics, and growth factors were continued. A diagnosis of hemophagocytic syndrome was considered, and intravenous cyclosporine was started. Despite aggressive transfusion support, minimal changes were noted in her pancytopenia and coagulopathy. Blood cultures were negative for bacterial, fungal, and mycobacterial pathogens. EBV serologies were notable for IgG EBV capsid protein and IgG EBNA antibodies, but negative for IgM EBV capsid antibodies. EBV DNA copy number in the blood was 27,800/ml, and the CMV DNA copy number was 16,500/ml. Tests for human herpesvirus 6 (HHV-6), Hepatitis C, Hepatitis B surface antigen, and human immunodeficiency virus (HIV) were negative.

On the third hospital day, she had increased retroperitoneal bleeding, as indicated by expansion of her flank ecchymosis and an increased red blood cell (RBC) transfusion requirement. She developed respiratory fatigue and required mechanical ventilation. Bronchoscopic examination was unremarkable, but lavage cultures were positive for *Stenotrophomonas maltophilia *and CMV. Ganciclovir was initiated. She was extubated the following day, but required bilevel positive airway pressure (BiPAP) and continued to have fever and a clouded sensorium.

**Figure 1:**
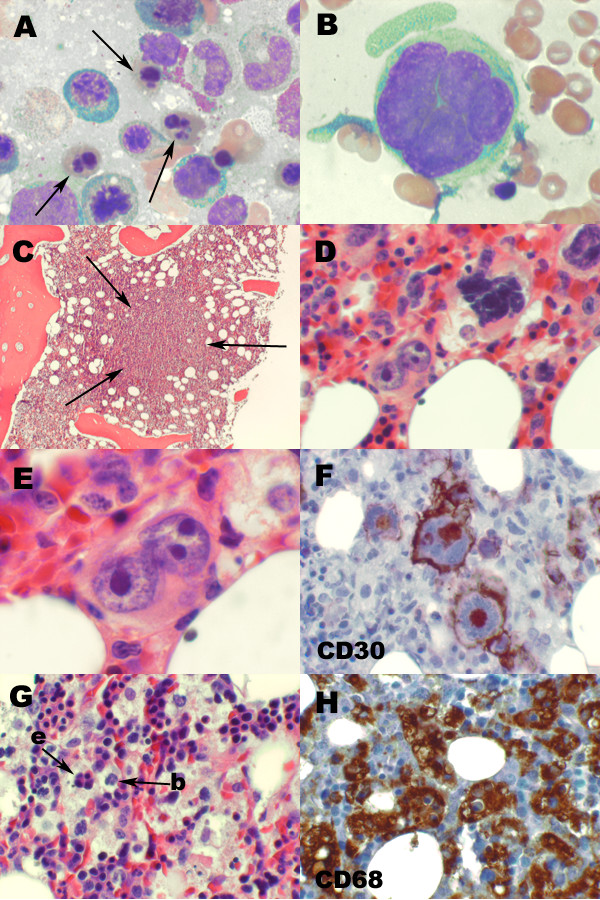
**Bone marrow**. (A) Aspirate smears show many dysplastic erythroid precursors with bizarre nuclear configurations (arrows) as well as (B) rare tumor giant cells (Wright Giemsa, original magnification ×1000). (C) Ill-defined tumor nodules efface the bone marrow architecture within the core biopsy (hematoxylin and eosin, ×100). (D) The tumor nodules contain many irregular mononuclear cells as well as scattered bizarre multinucleated tumor giant cells with hyperchromatic nuclei with coarse chromatin and macronucleoli (hematoxylin and eosin, ×400). (E) Occasional binucleated Reed-Sternberg like tumor cells are present (hematoxylin and eosin, ×1000). Immunohistochemical studies performed on the bone marrow core biopsy are consistent with classical Hodgkin lymphoma. (F) The tumor cells are strongly positive for CD30 expression with membranous and Golgi positivity (×400). (G) Within the bone marrow core biopsy, there is a conspicuous background histiocytosis with prominent hemophagocytosis (hematoxylin and eosin, ×400). Arrows indicate phagocytosed erythroid precursors (e) as well as an ingested band form (b). (H) An immunohistochemical stain for CD68 highlights abundant background histiocytes, many of which contain hematopoietic elements (×400).

A bone marrow aspirate revealed a hypercellular bone marrow containing maturing trilineage hematopoiesis with prominent megakaryopoiesis and hyperplastic dyserythropoiesis (Figure 1A). The erythroid series showed pronounced megaloblastoid change and abundant atypical erythroid precursors, including multinucleated normoblasts and many normoblasts with bizarre nuclear configurations (Figure 1A). Occasional large foamy macrophages containing other hematopoietic elements were noted. Rare very large atypical multinucleated cells with basophilic cytoplasm and separate oval nuclei with prominent nucleoli were also identified (Figure 1B).

The bone marrow core biopsy revealed a hypercellular marrow with background maturing trilineage hematopoiesis and a conspicuous increase in large histiocytes/macrophages, many of which contained hemosiderin and/or other hematopoietic elements, consistent with hemophagocytosis. In addition, tumor nodules were present, composed of large, irregular mononuclear cells with scattered, bizarre tumor giant cells containing hyperchromatic nuclei with coarse chromatin and prominent macronucleoli (Figures 1C and 1D). Occasional binucleated Reed-Sternberg (RS) cells were noted (Figure 1E). These tumor nodules occupied approximately 10% to 20% of the total marrow cross-sectional area within the core biopsy and were accompanied by a reticulin fibrosis. The tumor giant cells were strongly positive for CD30 (Figure 1F) and Ki67, but negative for other B-cell and T-cell markers, myeloid markers, CMV antigens and EBV latent membrane protein (LMP). Significant histiocytosis was also noted with phagocytosis of erythroid and myeloid cells (Figure 1G). CD68 staining revealed innumerable histiocytes/macrophages throughout the bone marrow interstitium, many of which contained numerous intact hematopoietic cells (Figure 1H). On the basis of these studies, she received a diagnosis of Stage IV HL with concurrent HLH.

She received a single cycle of dose-reduced adriamycin, bleomycin, vinblastine and dacarbazine (ABVD) chemotherapy for treatment of her HL. Two days later, she had a seizure of 2 to 3 minutes duration that involved her upper extremities and facial muscles. Her bilirubin rose to 316 μmol/liter (18.5 mg/dl), ferritin to 34,820 μg/liter, alanine aminotransferase (ALT) to 265 U/liter, and aspartate aminotransferase (AST) to 388 U/liter. Following a discussion with her health care proxy, comfort measures were instituted and she died the following day.

### Post-mortem pathologic studies

**Figure 2:**
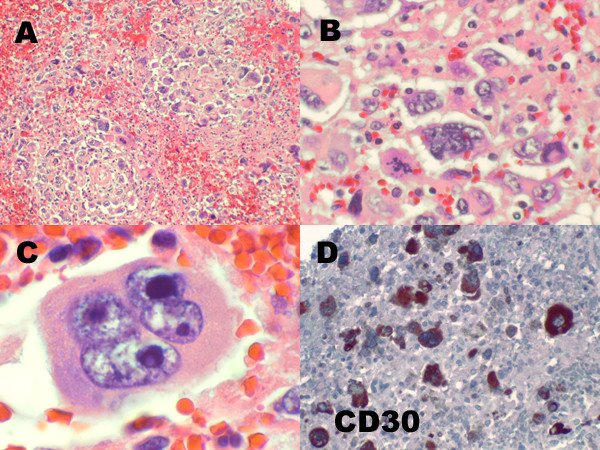
**Spleen at autopsy**. (A) Multiple tumor nodules efface the normal splenic architecture (hematoxylin and eosin, ×100). (B) The tumor nodules consist of clusters of numerous bizarre multinucleated tumor giant cells with irregular nuclei and coarse chromatin (hematoxylin and eosin, ×400). (C) Occasional classic Reed-Sternberg like tumor cells with macronucleoli are also present (hematoxylin and eosin, ×1000). (D) An immunostain for CD30 highlights the majority of the tumor giant cells (×100).

Gross examination revealed severe jaundice, petechial hemorrhages of the skin and gastric mucosa, mediastinal lymphadenopathy, and a hematoma over the left psoas muscle. Diffuse alveolar damage was noted in the lungs. Classical HL was observed in the spleen, liver, bone marrow, left kidney, and paratracheal lymph nodes (Figures 2A and 2B). The tumor infiltrates contained RS cells (Figure 2C), which were positive for CD30 (Figure 2D) but negative for other B-cell and T-cell markers and EBV LMP. Taken together, the bone marrow and autopsy findings confirmed a diagnosis of stage IV HL and HLH.

## Discussion

In acquired cases of HLH, the clinical course is rapidly progressive with multi-system organ failure often occurring within weeks of the initial diagnosis of the syndrome. In our patient, fulminant HLH was present for approximately 3 weeks before her death. The standard definition of HLH requires that at least 5 of 8 clinical criteria be met: fever, splenomegaly, peripheral cytopenias of 2 or 3 lineages, hypertriglyceridemia, elevated ferritin (>500 μg/liter), elevated soluble CD25 (sCD25), absent NK-cell activity, and histological evidence of HLH in bone marrow, lymph nodes, or spleen. Our patient had six of these: fever, splenomegaly, peripheral cytopenias of three lineages, hypertriglyceridemia, elevated ferritin, and histological evidence of HLH. Typical of HLH, she also had a coagulopathy, liver function test abnormalities, an elevated LDH, and CNS dysfunction.

It is possible that our patient had a chronic form of HLH. For 3 years before her admission, three of the diagnostic criteria for HLH were present: fever, cytopenias, splenomegaly. Additional laboratory studies to support the diagnosis were not obtained (triglycerides, ferritin, sCD25, and NK-cell activity). A bone marrow biopsy did not show hemophagocytic cells, but initial bone marrow biopsies are insensitive tests for the diagnosis of HLH [2]. In addition, there are few competing explanations for her cytopenias. In the absence of a clinically apparent malignancy and excluding HLH, the differential diagnosis for pancytopenia includes premalignant, inflammatory, infectious, genetic, and toxic causes (Table 2). Most of these could be ruled out on the basis of the history and laboratory studies. Furthermore, bone marrow did not show any premalignant, infiltrative or infectious processes, no toxins were involved, and an infection with parvovirus would have been self-limited.

**Table 2:** Non-malignant syndromes that can cause pancytopenia without HLH

**Infectious**
Parvovirus B-19
Visceral leishmaniasis

**Bone marrow failure**
Aplastic anemia
Myelodysplastic syndrome
Myelofibrosis
Vitamin B12 deficiency
Folate deficiency
Paroxysmal nocturnal hemoglobinuria
Sarcoidosis
Fanconi anemia
Gaucher disease
Niemann-Pick disease

**Inflammatory/Immune**
Transfusion-associated graft-versus-host disease
Evan's syndrome
Autoimmune lymphoproliferative syndrome
Aplastic anemia
Systemic lupus erythematosus

**Toxic**
Alcohol
Arsenic
Cyanide
Quinine
Methotrexate
Terfinabine
Tocainamide

Parvovirus and visceral leishmaniasis can also cause pancytopenia by inducing HLH. A diversity of infections can cause pancytopenia when associated with HLH. These are reviewed in Ref. 4 and at http://www.cdc.gov/ncidod/eid/vol6no6/fisman_refs.htm.

If our patient did have chronic HLH, what was the most likely cause? At the time of her death, she had three conditions associated with HLH: active EBV infection, active CMV infection, and HL. Her chronic HLH may have been the result of any of these, but we consider HL to be the most likely cause, since occult HL can exist for many years [12]. In contrast, acute EBV and CMV infections are associated with fever, pharyngitis, lymphadenopathy, and fatigue and would likely have been self-limited. EBV antigens are commonly expressed by RS cells in patients with HL and HLH [13]. In our patient, the RS cells were negative for EBV LMP, indicating that her HL and EBV reactivation were independent disease processes. Treatment with azathioprine and steroids may have facilitated reactivation of EBV and CMV late in her disease course while partially treating her HLH and HL.

## Conclusion

We have described a case of acquired HLH that presented in a fulminant form following a 3-year prodrome that was consistent with a mild, chronic form of HLH. Chronic HLH should be considered in the differential diagnosis of fever, splenomegaly and pancytopenia.

## Abbreviations

ALT: alanine aminotransferase; ANA: antinuclear antibody; AST: aspartate aminotransferase; BiPAP: bilevel positive airway pressure; CMV: cytomegalovirus; CT: computed tomography; EBV: Epstein-Barr virus; GI: gastrointestinal; Hb: hemoglobin; HHV-6: human herpesvirus 6; HIV: human immunodeficiency virus; HL: Hodgkin Lymphoma; HLH: hemophagocytic lymphohistiocytosis; LMP: latent membrane protein; MCV: mean cell volume; RBC: red blood cell; RS: Reed-Sternberg cell; sCD25: soluble CD25; WBC: white blood cell count

## Consent

Written informed consent was obtained from the patient for publication of this case report and any accompanying images. A copy of the written consent is available for review by the Editor-in-Chief of this journal.

## Competing interests

The authors declare that they have no competing interests.

## Authors' contributions

KC and SD analyzed and interpreted patient data and cared for the patient. WK and EB analyzed and interpreted the bone marrow and autopsy studies. DS performed the autopsy. EB prepared the figures. KC, EB and SD wrote the paper. All authors read and reviewed the final manuscript.

## References

[B1] Favara BE Hemophagocytic lymphohistiocytosis: a hemophagocytic syndrome Semin Diagn Pathol 1992 9 63 74 1561489

[B2] Janka G Zur Stadt U Familial and acquired hemophagocytic lymphohistiocytosis Hematology Am Soc Hematol Educ Program 2005 82 88 16304363 10.1182/asheducation-2005.1.82

[B3] Henter JI Samuelsson-Horne A Arico M Egeler RM Elinder G Filipovich AH Gadner H Imashuku S Komp D Ladisch S Webb D Janka G Histocyte Society Treatment of hemophagocytic lymphohistiocytosis with HLH-94 immunochemotherapy and bone marrow transplantation Blood 2002 100 2367 2373 10.1182/blood-2002-01-0172 12239144

[B4] Fisman DN Hemophagocytic syndromes and infection Emerg Infect Dis 2000 6 601 608 11076718 10.3201/eid0606.000608 PMC2640913

[B5] Billiau AD Roskams T Van Damme-Lombaerts R Matthys P Wouters C Macrophage activation syndrome: characteristic findings on liver biopsy illustrating the key role of activated, IFN-gamma-producing lymphocytes and IL-6- and TNF-alpha-producing macrophages Blood 2005 105 1648 1651 10.1182/blood-2004-08-2997 15466922

[B6] Kikuta H Sakiyama Y Matsumoto S Oh-Ishi T Nakano T Nagashima T Oka T Hironaka T Hirai K Fatal Epstein-Barr virus-associated hemophagocytic syndrome Blood 1993 82 3259 3264 8241498

[B7] Kluin-Nelemans JC Kluin PM Bieger R A 26-year-old man with Hodgkin's disease and rapidly progressive pancytopenia Ann Hematol 1993 67 49 56 10.1007/BF01709667 8334200

[B8] Kojima H Takei N Mukai Y Hasegawa Y Suzukawa K Nagata M Noguchi M Mori N Nagasawa T Hemophagocytic syndrome as the primary clinical symptom of Hodgkin's disease Ann Hematol 2003 82 53 56 12574967 10.1007/s00277-002-0572-2

[B9] Dawson L den Ottolander GJ Kluin PM Leeksma O Reactive hemophagocytic syndrome as a presenting feature of Hodgkin's disease Ann Hematol 2000 79 322 326 10.1007/s002779900130 10901612

[B10] Chim CS Hui PK Reactive hemophagocytic syndrome and Hodgkin's disease Am J Hematol 1997 55 49 50 10.1002/(SICI)1096-8652(199705)55:1<49::AID-AJH10>3.0.CO;2-1 9136919

[B11] Korman LY Smith JR Landaw SA Davey FR Hodgkin's disease: intramedullary phagocytosis with pancytopenia Ann Intern Med 1979 91 60 61 464459 10.7326/0003-4819-91-1-60

[B12] Garcia-Carbonero R Paz-Ares L Arcediano A Lahuerta J Bartolome A Cortes-Funes H Favorable prognosis after late relapse of Hodgkin's disease Cancer 1998 83 560 565 10.1002/(SICI)1097-0142(19980801)83:3<560::AID-CNCR26>3.0.CO;2-T 9690550

[B13] Menard F Besson C Rince P Lambotte O Lazure T Canioni D Hermine O Brousset P Martin A Gaulard P Raphaël M Larroche C Hodgkin lymphoma-associated hemophagocytic syndrome: a disorder strongly correlated with Epstein-Barr virus Clin Infect Dis 2008 47 531 534 10.1086/590152 18611160

